# Retraction: Tranexamic Acid in Pregnant Women With Placenta Previa: A Double-Blind, Multicenter Randomized Clinical Trial

**DOI:** 10.7759/cureus.r200

**Published:** 2025-11-11

**Authors:** Shahla Alalaf, Ariana K Jawad, Namir G Al-Tawil, Kawa F Dizaye, Sileem A Sileem, Noor Elebiary, Zanwer S Mahmood, Rozhan Y Khalil, Khalida M Amin, Nazdaar R Ali

**Affiliations:** 1 Obstetrics and Gynecology, Hawler Medical University, College of Medicine, Erbil, IRQ; 2 Obstetrics and Gynecology, Kurdistan Higher Council of Medical Specialties, Erbil, IRQ; 3 Community Medicine, Hawler Medical University, Erbil, IRQ; 4 Pharmacology, Hawler Medical University, College of Medicine, Erbil, IRQ; 5 Obstetrics and Gynecology, Al-Azhar University, Faculty of Medicine, Assuit, EGY; 6 Obstetrics and Gynecology, Ninewells Hospital, Dundee, GBR; 7 Obstetrics and Gynecology, Ministry of Health, Maternity Teaching Hospital, Erbil, IRQ; 8 Obstetrics and Gynecology, College of Medicine, University of Sulaimani, Sulaimaniyah, IRQ; 9 Obstetrics and Gynecology, University of Kirkuk, Kirkuk Medical College, Kirkuk, IRQ; 10 Obstetrics and Gynecology, Duhok Obstetrics and Gynecology Teaching Hospital, Duhok, IRQ

This article has been retracted by the Editors-in-Chief due to concerns over the timeline and feasibility of the study. The treatment effect is also questionable and lack of raw data upon request undermines the credibility of this article. The authors disagree with the decision to retract.

